# The Appropriateness of Medical Devices Is Strongly Influenced by Sex and Gender

**DOI:** 10.3390/life14020234

**Published:** 2024-02-07

**Authors:** Ilaria Campesi, Flavia Franconi, Pier Andrea Serra

**Affiliations:** 1 Dipartimento di Scienze Biomediche, Università degli Studi di Sassari, 07100 Sassari, Italy; 2 Laboratorio Nazionale sulla Farmacologia e Medicina di Genere, Istituto Nazionale Biostrutture Biosistemi, 07100 Sassari, Italy; franconi.flavia@gmail.com; 3 Dipartimento di Medicina, Chirurgia e Farmacia, Università degli Studi di Sassari, 07100 Sassari, Italy; paserra@uniss.it

**Keywords:** sex differences, gender differences, medical devices, safety profile, efficacy

## Abstract

Until now, research has been performed mainly in men, with a low recruitment of women; consequentially, biological, physiological, and physio-pathological mechanisms are less understood in women. Obviously, without data obtained on women, it is impossible to apply the results of research appropriately to women. This issue also applies to medical devices (MDs), and numerous problems linked to scarce pre-market research and clinical trials on MDs were evidenced after their introduction to the market. Globally, some MDs are less efficient in women than in men and sometimes MDs are less safe for women than men, although recently there has been a small but significant decrease in the sex and gender gap. As an example, cardiac resynchronization defibrillators seem to produce more beneficial effects in women than in men. It is also important to remember that MDs can impact the health of healthcare providers and this could occur in a sex- and gender-dependent manner. Recently, MDs’ complexity is rising, and to ensure their appropriate use they must have a sex–gender-sensitive approach. Unfortunately, the majority of physicians, healthcare providers, and developers of MDs still believe that the human population is only constituted by men. Therefore, to overcome the gender gap, a real collaboration between the inventors of MDs, health researchers, and health providers should be established to test MDs in female and male tissues, animals, and women.

## 1. Introduction: The Sex and Gender Gap—An Endemic Problem Induced by Men’s Power and Male Predominance in Research

According to the World Health Organization [1], medical devices (MDs) are essential for the safe and effective prevention, diagnosis, treatment, and rehabilitation of illness and diseases, and are largely marketed all over the world [2]. There are numerous types of MDs. For example, more than >500,000 types ranging from syringes, hearing aids, stethoscopes, and shoes to ventilators, catheters, stents, defibrillators, and X-ray equipment are on the European market [3].

In 2001, the Institute of Medicine indicated that sex differences exist and can exert their influence in many aspects of health and care and, therefore, they should be considered a relevant variable in biomedical research and clinical practice [4]. Those authors tried to dispel the belief that the man’s body was the norm. This is a belief that has accompanied humanity since ancient times [5], and numerous individuals including health providers continue to uphold/sustain it [6], rendering women invisible. Paradoxically, all this happens while women outnumber men in all countries except for China and India [7], and, more importantly, the prevalence of women is greater after the age of 50, and this is also the group that requires more care and MDs.

The individuation and description of sex differences led to the birth of sex-based biology and two diverse but interacting concepts: “sex” and “gender”. They have a distinct meanings, although they are strongly interrelated, forming Gordian nodes; thus, they are not mutually exclusive [8]. Gender, sex, and their interactions and intersections may trigger epigenetic changes resulting in physiological modifications that may impact health, etiology, and the presentation of diseases, their outcomes, and treatment responses [9,10]. It is important to underline the fact that biological sex falls on a spectrum that encompasses many sexes and those who are intersex [11]. Due to the lack of data on the above populations [12], from here on we will refer only to women and men. 

Interest in sex and gender differences is growing, and an ever-increasing number of differences are found in morphology, physiology, diseases, and treatments (Table 1); unfortunately, they are still little-studied and -published, especially in scientific journals with low impact, as evidenced by a bibliometric analysis of over 11 million articles [13]. This is a result of several impediments. In particular, some researchers still believe today that men and women are similar, others know there are sex differences but are not able to complicate their experimental design by enrolling women [14]. Paradoxically, others believe that women are lucky not to be enrolled given the risks in the initial stages of registration trials [14]. Others state that the low enrollment of women depends on ethical reasons, such as the fear of causing harm to a fetus, forgetting that women in menopause are not fertile. Finally, many women are reluctant to participate in clinical trials, and accounting for sex and gender differences leads to a substantial increase in economic cost, as trials need a larger number of subjects [15,16]. This phenomenon may lead to undetected or unrecognized efficacy and safety profiles, which are discovered after the introduction of MDs to the market.

Without “female” data, it is impossible to know whether the results obtained in men also apply to women, and this may have wide-reaching consequences [10,17]. For example, women with dementia receive worse medical treatments than men [18]. In the United Kingdom, it was evidenced that numerous women suffered harm because of poor healthcare [19], probably because health professionals were forced to extrapolate women’s medical recommendations from those based on the Vitruvian man [20]. A Food and Drug Administration (FDA) report from 2019, which examined 340,000 reports of device-related adverse effects including deaths, evidenced that they prevailed more in women (67%) than in men [21]. To overcome this situation, in 2014 and 2019, the FDA prepared specific guidelines [22,23].

As already mentioned, especially in women and other minorities, MDs may not have been sufficiently tested for efficacy and safety before their introduction to the market [2]. For example, surgical meshes, which are largely used in hernias, uropelvic surgery, and to treat incontinence, were not tested on women before their introduction to the market [24,25], although, worldwide, there are 20 million annual hernia surgeries on women [2]. The FDA’s warnings about uropelvic meshes came after numerous women reported side effects [24]. In addition, the first artificial hearts were more dangerous for women than for men because they were too large for women’s rib cages [26,27]. Thus, MDs induce more side effects in women and other minorities than in men [24,28,29].

Here, we try to highlight how sex and gender influence the efficacy and safety of MDs, including simple ones such as syringe needles and surgical masks and the more complex ones, offering a series of examples based on the clinical relevance of sex and gender differences. This review aims to avoid perpetuating the under-enrollment of women in clinical investigation, to increase the sex stratification in research, discovery, design, and development, and to increase stakeholder awareness of the importance of using a sex–gender approach to ensure more rigorous sex–gender data in the field of MDs. Data from female animals and women are a prerequisite for supporting equitable health either in general or in the MDs ecosystem [23].

**Table 1:** Some examples of sex and gender differences.

Parameters	Sex and Gender Differences
Body dimension (both in weight and height)	+men
Bone mass	+men
Bone health	+reduction in bone mass in women after menopause
Skeleton muscle	+men
Presence of muscle Type 2 fast-twitch fibers	+muscle
Subcutaneous fat	+in women
Hearing loss	+men
Cochlear length	+men
Vision alterations	+women
Skin pore size	+men
Gastrointestinal motility	+men
Heart mass and volume	+men
Heart rate	+women
QT interval	+longer in women
Right ventricle ejection fraction	+women
Left and right ventricular stroke volume	+men
Cardiac output	+men
Resting blood pressure	+ men
Systolic blood pressure	+men
Fibrinogen	+women
Abdominal aortic aneurysm	+men
Liver dimension	+men
Alcohol clearance	+men
Susceptibility to alcohol injury	+women
Alcoholic liver disease prevalence	+men
Autoimmune hepatitis prevalence	+women
Hepatocellular carcinoma prevalence	+men
Liver cirrhosis prevalence	+men
Drug-induced liver injury prevalence	+women
Liver cholesterol metabolism	+women
Kidneys	larger in men
Glomerular filtration rate	+men
Kidney replacement therapy	+men
Chronic kidney disease prevalence	+women
Prevalence of end-stage kidney failure	+men
Urethra length	+men
Urinary infection prevalence	+women
Type 2 diabetes complications	+women
Insulin sensitivity	+women
Laryngeal lesion prevalence	+men
Lungs, airways, and lung diffusion capacity	+in men even if corrected for height
Forced vital capacity	+women
Diaphragm length	+men
‘Pyramidal’ lung geometry	+men
‘Prismatic’ lung geometry	+women
Chronic obstructive pulmonary disease	+women
Autoimmune disease prevalence	+women
Alzheimer’s disease + other dementia prevalences	+women

Data from [10,30,31,32,33,34,35,36,37,38,39,40,41,42,43,44,45,46,47,48,49].

## 2. MDs’ Research, Design, and Developmental Process and Sex and Gender Aspects

Although sex and gender experts demonstrate extensive attention to sex and gender factors, these aspects are still neglected in MDs [15,50]. The exclusion of sex- and gender-related factors have been justified by the actors of the process (industry and investors etc.) due to the extra work and resources, because sex and gender approaches require an increase in research costs and an elevation of the product price, as stated by Vijayasingham and collaborates [50].

**Figure 1:**
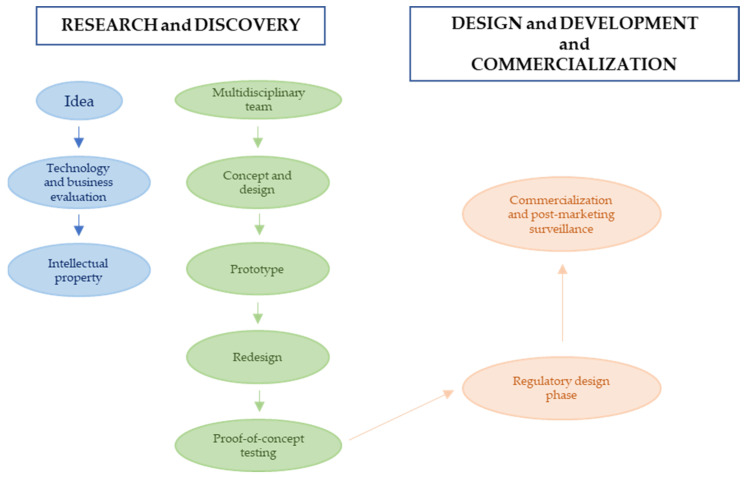
Stages of the research, discovery, design, and development process of MDs.

The research and developmental process of MDs (Figure 1) is a complex and expensive procedure that can also present several pitfalls [51], as evidenced by the fact that only 6% of designed MDs reach the market [52].

Importantly, the research, design, discovery, and developmental process of MDs imply an integration of medicine and engineering. Hence, a multidisciplinary team with expertise in engineering and design, human factors and usability engineering, clinical and scientific knowledge, regulatory affairs and quality assurance, and intellectual property should be involved to obtain an MD with the best possible quality. Importantly, MDs’ development also depends on the risk associated with them [53]; therefore, the hazard analysis should be considered a major component and operational requirement in human–device interfaces and services. In other words, the MDs industry should become aware that the characteristics of patients are seldom universal. 

### 2.1. Technologies, 3D Scanning and Artificial Intelligence (AI), That Help Designers Consider Sex and Gender Aspects If Appropriately Used 

Notably, there are technologies such as 3D scanning and 3D printing that are used for creating ergonomic prosthetic devices and dental implants that allow for the customization of MDs on single patient, for example adapting an MD to anatomical differences that characterize the two sexes [54]. 

AI and machine learning can support the MDs process and can simplify and mitigate risks and cut costs [55]. Relevantly, the algorithms of AI can perpetuate or decrease the sex and gender bias depending on how they are developed, without removing bias and confounding factors or integrating sex and gender differences [56]. Unfortunately, despite the influences of sex and gender on health and medicine, most biomedical AI technologies do not consider sex and gender [56,57]. In addition, fewer women are included in AI studies, including those focused on digital biomarkers. For example, a study assessing digital biomarkers for Parkinson’s disease involved only 18.6% women [56]. If an algorithm is built with data mainly obtained from men it will be more accurate for men than for women. Thus, AI must consider sex and gender as variables to build MDs appropriate for all.

### 2.2. Are Natural and Synthetic and Biomaterials Sex- and Gender-Susceptible?

Even though knowledge about sex and gender differences has steadily increased in the last two decades, sex and gender differences in biomedical engineering are still neglected, although many agencies including regulatory ones consider sex as a biological variable from several perspectives [58,59]. However, these perspectives have been scarcely adopted by the biomedical engineering community. A survey (2017–2022) found that only 53–65% of engineered tissues considered sex and gender aspects [60]. Worse results are obtained when the community of biomaterials is considered, because only ∼4% of studies reported the cell sex, and none included it as a variable of interest [60]. 

It seems appropriate to make a brief mention of materials, because male and female cells may respond differently to the natural and synthetic biomaterials that are used to repair or substitute injured tissues or organs or mimic an organ’s functions [61,62]. However, only a minority of biomaterial studies (3.7%) are performed on cells. In addition, the vast majority of studies on biomaterials did not report any demographic information that could help develop personalized biomaterials [61]. Given the influences of sex and gender on human cells, organs, and tissues [47,63], the incorporation of sex and gender into engineered biomaterials is strongly recommended. For example, according to Lock and coworkers [64], the possibility of having female and male engineered myocardial tissues could help to elevate our knowledge on the effect of exogenous exposure to sexual hormones, and this could also improve myocardial health in transgender populations. Sex differences are not limited to cells but also involve micro-environments [60]. Indeed, physical signals (for example, size, shape, porosity, dimensionality) coming from the extracellular matrix are sex-dependent and may be affected by sex hormone stimulation [60].

### 2.3. Biomedical Sensors for Healthcare Monitoring 

Numerous barriers exist in chronic disease prevention, monitoring, and early intervention. Given the increase in the prevalence of chronic diseases, especially in the elderly, there is a need for continuous monitoring and long-term care. The use of glucose biosensors, for example, has improved the quality of life of millions of diabetic patients across the world [65]. Thus, the use of medical sensors is a way to facilitate disease monitoring, as testified to by the increasing use of sensors. Healthcare monitoring sensors represent, in fact, an exponentially growing research field and market with important financial investments. Biosensors can be classified according to the kind of signals/analytes they aim to monitor. It is important to note that the majority of biomedical sensors used for health monitoring are not inherently designed according to aspects of sex and gender [66]. Therefore, sex and gender perspectives are missing. For example, the majority of heart rate monitoring sensors do not consider sex differences in cardiac rate and variability when analyzing and interpreting data [67,68,69,70,71]; although the average heart rate ranges from 70 to 72 and 78 to 82 beats/min in men and women, respectively, and differences may increase during exercise [72]. In addition, stress and emotional states may affect the heart rate variability of women and men differently. For example, 24 h of sadness increases and decreases the heart rate variability in women and men, respectively [73]. 

Adopting sex and gender approaches to biosensors, especially wearable sensors, is also needed, to increase comfort and compliance with their use [66]. Wearable devices represent an innovative method of clinical diagnostics, as they exploit various physical, chemical, and biological factors to extract physiological information in real time and continuously, without using invasive methods [74]. Their design and esthetics (see smartwatches or fitness trackers), can sometimes differ to accommodate different wrist sizes or personal preferences [75,76,77]. A recent review that focused on electronic design for wearable devices indicated that anatomical and physiological factors must be considered [66]. However, a sex- and gender-sensitive approach to design should consider the behavioral and sociological differences between genders, because design can show implicit biases which may negatively impact on female and gender non-conforming people’s user experience [66].

Although some papers consider usability, comfort, wearability, etc., according to sex and gender, a global sex–gender approach is missing, because other factors are necessary, such as sensors, communication technologies, etc., which have not received enough attention [66]. Historically, in fact, designers are predominantly young men, and this implicitly leads to numerous prejudices in all phases of design [66]. For example, it is worth reporting that aspects of sex and gender design are less often present in women’s clothing because they are missing pockets suitable for smartphones [66]. 

Furthermore, fitness- and activity-tracking sensors consider factors like body size, weight, and stride length to estimate metrics such as calories burned or distance traveled. In these cases, there might be slight variations, based on sex differences, in body composition or average physical characteristics [68,78,79]. 

Numerous sensors have evidenced the sex and gender differences in sleep patterns and sleep-related pathologies, and these have been reviewed in Bublitz [80]. It is important to underline that sex and gender differences depend also on whether a person is pre-menopause or post-menopause, or on the use of hormone replacement therapy [81]. Monitoring, mainly based on polysomnography and movement studies, indicates that women have less access to this technology [82].

Moreover, other objects of scientific interest, even if they are more complex to realize, are biosensors for the detection of analytes that occur in biological fluids to detect and quantify specific substances, such as biomarkers [83]. Sex and gender are not usually considered in the design or functionality of these biosensors, which are apparently gender-neutral, but it is well known that androcentrism prevails in prevention and medicine [10], with men being the gender-neutral standard [84]. Moreover, in this regard, it should be remembered that the human metabolome presents numerous sex and gender differences [85,86].

A final consideration must be made regarding the ease of access to these technologies, which are often designed without adequate consideration of sex and gender preferences [87]. In fact, sex and gender influence both the private use of health monitoring applications and the acceptance and use of health technologies in clinical settings. Therefore, it already appears to be important to use a sex and gender perspective in the design phases of digital devices and services for healthcare and lifestyle applications.

## 3. Access to MDs 

Retrospective investigations evidenced the sex and gender disparities in the access to medical technology, including MDs [2]. The access to MDs depends on numerous factors which can also differ greatly from each other, such as access to digital devices with good connectivity, culture and ethnicity, age, poverty, sex and gender, etc. [88]. MDs should be accessible to all for appropriate prevention and care and to observe the principle of equity in care delivery, which means giving equal access to the whole population, irrespective of residence, gender, caste, economic strata, or other factors [89]. 

It has been described that women have less access to cardiac, renal, and urinary MDs. For example, women with heart failure receive fewer left ventricular assist devices (LVADs) [90,91,92]. The disparity of access to cardiovascular MDs is confirmed by very recent studies, which have evidenced that the use of LVADs in patients with heart failure with a reduced ejection fraction was smaller in women [93], such as the use of catheter ablation for rhythm control in atrial fibrillation [94]. Interestingly, it has also been evidenced that the effect of sex and gender on access to catheter ablation and LVADs intersect with racial and ethnic aspects. De Silva and coworkers evidenced that pacemakers, cardioverter defibrillators, and resynchronization therapy are less used in women than in men, independent of age or comorbidities [95]. In addition, women receive less invasive mechanical ventilation in comparison to men [96], as observed during general anesthesia [97] in critically ill patients [98,99], as well as less dialysis [100]. Furthermore, a German study highlighted that the prevalence of the continuous use of blood glucose monitors depends on age, sex, and gender [101]. In short, initially, male and female children have the same prevalence, while girls and adult women use them more than boys and adult men [101]. However, the influence of sex and gender on metabolic control is not unique, as some authors have found it to be similar between men and women [101], while others discovered that more men reached values of glycated hemoglobin ≤ 7% [102].

The lower use of MDs among women may reflect a sex and gender bias and requires further research. In addition, their decreased access to MDs is a subtle form of discrimination linked to sex and gender that can be considered a form of chronic cumulative stress, which can affect the health of an individual, causing further barriers to prevention and care access [103].

## 4. The Safety of MDs for Health Operators 

In the health system, female workers are better represented at all levels than in other sectors; however, sex and gender biases and inequities are still present [104]. 

Despite female predominance, which, globally, determines a greater use of MDs by female health carers, their safety profiles, especially in female workers, are often under-reported [105]. For example, the morphological sex differences in hand width, strength, height, arm span, etc., strongly indicate the need for instruments designed for single-sex anatomy to avoid ergonomic problems arising from the chronic use of instruments that are not adapted to health carers’ morphological features. A recent meta-analysis related to Traditional Laparoscopic Surgery evidenced that female surgeons were slower and reported more pain (neck and shoulder) than their male counterparts [106]. 

Finally, 5.6 million healthcare providers have been injured by needlesticks, bistoury, etc., which can expose them to infective diseases such as hepatitis B and C and HIV [107,108].

In view of fact that sex and gender medicine involves not only the patient but also healthcare providers [109], it therefore seems appropriate to alert the MDs industry that it is time to stop to designing MDs based on male characteristics. Because, in fact, it is time to consider the sex and gender of care providers in the design and production of MDs to develop ergonomic MDs either for men or women.

## 5. Some Examples of Sex and Gender Influences on the Safety and Efficacy of MDs in Patients 

This section reports a heterogeneous series of MDs that may have different effects on men and women. The choice of examples is based on highlighting the fact that sex–gender bias is practically widespread, as confirmed by the fact that in the last 40 years, in the USA, some MDs have been removed from the market as they were reported/proven to be more harmful to women [110]. 

### 5.1. Syringes and Needles: Are Buttock Injections Truly Intramuscular, Especially in Women?

The intramuscular administration (IM) of medications is quite common and the gluteal and deltoid regions are the most common sites of administration. Given the sex difference in subcutaneous fat (>in women than in men), women, especially those who are overweight or obese, require longer needles than men to avoid a loss of drug activity due to an incorrect site of injection (subcutaneous fat instead of skeletal muscle). Obese men (body mass index > 35) may also require longer needles [111,112]. During emergencies, this could have serious consequences, especially when these require self-administration of drugs, such as the self-administration of epinephrine for the treatment of anaphylaxis [113]. In fact, the treatment failure of intramuscular injection is 54.4% and 5% in women and men, respectively [114]. In conclusion, if the needle length is not adjusted for the individual, the rate of successful IM injections is lower in women than in men [115], and this could affect the efficacy and safety profiles of these treatments. 

### 5.2. Venus and Mars See Differently 

Two thirds of all blind people in the world are women [116], and numerous vision alterations such as age-related macular degeneration, thyroid eye disease, normal-tension and angle-closure glaucoma, cataracts, and dry eye diseases affect more women than men [30,31,32]. The impact of sex and gender on vision is scarcely studied [31], and many studies that include men and women did not stratify the results according to sex [117], and this is reflected in MDs.

To a degree, both exogenous and endogenous estrogen may play a crucial role in the genesis of these sex differences [118]. For example, pregnancy decreases mean intraocular pressure [119] and changes the corneal morphology (thickness) and function (corneal perception) of female eyes [120]. Interestingly, specific female treatments (menopausal hormone replacement therapy) can influence corneal function [121]. Notably, in Japan, corneal refractive surgery is contraindicated in pregnancy [120]. 

Ocular sex–gender differences influence both non-invasive (glasses and diagnostic instruments) and invasive (contact lens and their care products, implantable devices, etc.) MDs. For example, worldwide, women use more contact lenses [122] and suffer more adverse effects (contact dermatitis, bacterial infections, dry eye disease, ocular surface discomfort, and other serious conditions) than men [123,124]. In addition, the constant use of topical cosmetics may influence the adverse effects of contact lenses [32,125]. 

Thus, it is mandatory to adopt sex- and gender-sensitive approaches to investigate eye MDs, including those that are more simple, such as artificial tears and contact lenses. 

### 5.3. The Sex of the Human Respiratory System 

The respiratory system’s structure and functions depend on a person’s sex, as already described by numerous authors [33,34,35]. In particular, sex differences are visible in the larynx through the so-called male Adam’s apple [33]. Some of them [126,127] start in a prenatal period and continue for all of one’s life [33]. It has been noted that some are influenced by height or lung volume [33], which differ between sexes. Importantly, in women, lung function is influenced by sexual endogenous and exogenous hormones [128]. For example, forced expiratory volume and forced vital capacity are increased during the luteal phase of the menstrual cycle [128].

However, these differences are neglected in the design, projection, and building of MDs. For example, supraglottic airway devices, which can be used without a pharmacological neuromuscular block [129], induce postoperative sore throats and hoarseness more frequently in women than in men [130,131]. 

Wearing a face mask have a beneficial effect in the prevention of respiratory infections, as also observed in recent COVID-19 pandemic [132]. To have the best prevention it is very important to have a comfortable mask that fits well [133]. However, many face masks are designed following the paradigm “one-size-fits-all”, even though male and female human faces display sex-specific morphological features that depend on age [134,135]. Considering that the majority of protective masks are designed for a male face, it is easy to imagine that women are less protected by these MDs as they do not perfectly fit with female facial features [136].

The administration of drugs via the respiratory route is essential for asthmatic and chronic obstructive pulmonary disease (COPD), and the choosing of the right inhaler requires moving towards personalized medicine. Real-world data evidenced that if a specific inhaler device is unrelated to the characteristics of asthmatic and COPD patients [137,138] it may increase non-adherence to treatment [139]. 

The sex of patients seems to have a role in the correct use of inhalers. A past investigation once evidenced that 43% and 4% of men and women used the correct inhaler technique, respectively [140]. A more recent study evidenced that the correct use of an inhaler depends on sex, disease, and the type of inhaler [141]. For example, female asthmatic patients make more mistakes with dry-powder inhalers, while COPD soft mist inhalers also produce more mistakes when used by female patients. Indeed, errors were major in female COPD patients with regard to their use of soft mist inhalers, whereas with metered-dose inhalers there were no sex–gender differences observed in patients with COPD [141]. 

It is evident that health professionals and researchers, including engineers, would do a better job if they took sex and gender into consideration. 

### 5.4. The Urinary System: A Well-Known Difference

It does not take a doctor to understand that the external urinary system is very different in women and men. However, numerous sex differences also modulate the internal urinary system, such as urethra length, renal structure, and function, and they have already been described [36,37]. Despite this, women receive less evidence-based medicine than men for the management of their urinary system [142]. For diagnosis, urine dipsticks give more false positives in women (8.7%) than in men (5.6%) [143]. The performance of automated urinalysis is better for men than for women [143]. 

Hemodialytic patients need permanent vascular access through an arteriovenous fistula, or arteriovenous prosthetic graft, or a tunneled central venous catheter. The latter is used more in women than in men [144]; nevertheless, mechanical and thrombotic complications are higher in women than in men [145]. In addition, some authors maintain that being a woman is a risk factor for line dysfunction with tunneled central venous catheters [146].

Obviously, there are both male and female urinary catheters. In adults and pediatric-age patients [147,148], urinary catheters lead to infections in the urethra, bladder, or less commonly in the kidneys, especially in women. The reason for this is not clear but it is important to underline that, in absence of a catheter, urinary infections exhibit one of the most prominent sex disparities among infectious diseases, with premenopausal women being 20–40 times more likely to have an infection than men of the same age [149]. This could depend on morphological differences such as the shorter distance between the anus and the urethral opening in women than in men and/or sexual hormones which can regulate immune system [149]. Catheters are, in fact, responsible for about 75% of hospital urinary infections, with the risk being higher with indwelling catheters [150]. Conversely, non-infective side effects prevail in men [151]. 

In this context, it is important to remember (a) the huge market value of catheters (at USD 3.4 billion for years), (b) the fact that catheter use is highly inappropriate, especially in women, and (c) that there has been little innovation in this area in the last 80 years [150,152]. 

### 5.5. The Cardiovascular (CV) System, Where Sex–Gender-Based Medicine Originated 

Although there are some large studies that have exclusively enrolled women, women are still under-enrolled cohorts in CV diseases, with large variation among CV diseases [153]. Currently, numerous sex and gender differences are reported in the CV system. These differences are extensively and expertly described in many articles and reviews [38,39,40,41]. 

Many types of CV MDs are available, but they are not built for women [41]; therefore, it is not surprising that sex and gender differences have been described in clinical outcomes and safety profiles with the use of numerous MDs [28]. Some examples are given below.

Stents and grafts: Stents improve survival and decrease adverse events in an acute myocardial infarction both in men and women, but have a lower safety profile in women. Women have a greater risk of bleeding induced by antithrombotic drugs and vascular complications than men [22]. Further, women have also experienced more major adverse cardiovascular events [154]. However, the two registries DELTA (Drug-Eluting Stents for Left Main Coronary Artery Disease) and DELTA-2, which include 27% of women, demonstrate that women were older, and more often had diabetes and chronic kidney disease than men. Overall, multivariable analysis has evidenced that sex and gender did not appear to be independent predictors for the primary outcome in revascularization for left main coronary artery disease [155]. 

Concerning grafts, which are declining in women but not in men, coronary artery bypass grafting seems to be associated with a lower risk of primary endpoint myocardial infarction, cerebrovascular accidents, and all-cause death in comparison with percutaneous coronary intervention [156]. However, women seem to present more adverse outcomes (bleeding and death) after undergoing coronary artery bypass grafting [22,157,158,159]. In the same way, women receive fewer multiple arterial grafts than men and the right internal thoracic artery is more underused in women than in men, due to the limited randomized evidence in support of its clinical benefits [160].

Mechanical Circulatory Support (MCS): MCS includes left and right ventricular assist devices (LVAD and RVAD) [41]. MCS offers support to patients before percutaneous coronary intervention and transplantation, cardiogenic shock, heart failure, chronic end-stage heart failure, and acute hemodynamic embarrassment [41]. Again, this support is less studied in women (20% to 25% of participants) [161], who receive less MCS [162]. Nevertheless, data show greater beneficial effects in women compared to men from temporary and durable MCS [41]. In particular, the use of this support before percutaneous coronary intervention is linked to greater survival in women than in men [41], but these results are not univocal, because another study shows that the survival at 30 days is similar in both sexes [41]. A registry study, where women presented more comorbidities, showed that hospital mortality, myocardial infarction, stroke, and ejection fraction improvement were similar between sexes, but women have more multivessel revascularization and bleeding than men [163]. In line with previous results, women can develop more vascular complications with Impella devices, probably due to/as a result of their smaller arterial size, and, with an LVAD, women can experience more thromboembolic events [41]. 

Probably, because of the smaller size of their rib cage, women appear less likely to have an earlier durable MCS and those that do have a lower safety profile when there are major adverse cardiac events compared to men [162]. In particular, women are at greater risk of both cardiovascular and neurological complications [22]. By reinforcing the concept of the scarce attention paid to the differences between men and women in some devices, such as the Tandem Heart device (TandemLife, Pittsburgh, PA, USA), sex differences were not investigated in their outcomes [164]. A study that included 1237 patients (27% women) with a similar Preprocedural SYNTAX score evidenced no sex or gender differences in 90-day major adverse cardiac and cerebrovascular events such as vascular complications requiring surgery, major bleeding, or acute limb ischemia being (after propensity matching and multivariable regression), the sex differences were limited to immediate PCI-related complications [165]. 

Pacemakers: In men, the main indication for pacemaker implantation is an atrioventricular block, while in women the main indications are sick sinus syndrome and atrial fibrillation with brady-arrhythmias [28]. In elderly patients (>80 years), dual-chamber pacemakers are more used in men than in women [28]. However, amiodarone-induced brady-arrhythmias are more frequent in women and require pacemaker implantation in women but not in men [28]. 

The survival at 30 years on from the implantation of permanent pacemakers seems to be higher in women than in men but no univocal data are available on life quality [28]. Periprocedural side effects (pneumothorax, cardiac perforation, and pocket hematoma) occur more frequently in women than in men [92,166] and, vice-versa, infective complications seem to be more frequent in men, but the data are non-univocal [28]. One reason could be the fact that women receive a pacemaker at an older age and for a longer time, given their longer life expectancy [92,166].

Cardiac resynchronization therapy (CRT): Clinical trials for CRT were performed mainly in men, but CRT is more effective in nonischemic cardiomyopathy, which prevails in women [28]. Thus, women seem to benefit more than men from CRT [28]. In women, the left bundle-branch block and shorter QRS interval could be criteria for treatment [28,167]. 

Implantable cardioverter-defibrillators (ICDs) and cardiac resynchronization therapy defibrillators (CRTDs): Very few women (less than 30%) have been enrolled in a study evaluating ICDs and they are less used in women [28,167], although women have longer daily use of the device [168]. A very recent paper published in *JAMA* using ICDs or CRTDs (4506 patients: 1075 women and 3431 men) evidenced that women received more CRTDs than men [169]. Interestingly, with CRTDs, women had more benefits than men, especially with left bundle-branch block and with a shorter QRS interval [170,171]. Finally, women who received ICDs had less first ventricular arrhythmias and lower ventricular tachyarrhythmia [169]. Although some data suggested a lower efficiency of these devices in women, recent data performed with a large number of women show that women and men have similar outcomes, even if more symptoms of advanced heart failure are more present in women than in men [167]. Interestingly, physical activity is higher in men than in women, in patients with a ICD/CRT-D [172,173,174,175]. However, other authors evidenced comparable outcomes in both women and men [176,177].

Regarding safety profiles, the risk of ventricular tachycardia or ventricular fibrillation is higher among women than among men [168]. In secondary prevention cohorts with ICDs, women present more ventricular fibrillation and have a higher re-intervention rate than men [167]. A lower safety profile was also found in women with ischemic cardiomyopathy and congenital/inherited heart diseases, which present a larger burden for ventricular arrhythmias, than in men [168]. 

Mitral valve (MV) replacement: Recently, it has been shown that there are numerous sex and gender differences in mitral regurgitation outcomes based on differences in morphology and pathophysiology, which may slow down diagnoses and treatments in women [178]. Female patients have a higher prevalence of rheumatic mitral valve disease and mitral valve prolapse, while ischemic mitral regurgitation prevails in male patients. This implies that patients with rheumatic disease are less likely to have a successful surgical mitral valve repair [178]. 

The main treatments are MV surgery and surgical MV repair, depending on the clinical situation. A very recent and exhaustive review has examined the sex differences in MV replacement, and concluded that not all disparities can be mitigated through the validation of sex-specific diagnostic criteria and treatments [178]. However, a recent paper evidenced that men and women have similar survival rates after transcatheter tricuspid valve interventions [179]. The Transcatheter Tricuspid Valve Therapies (TriValve) registry evidences that tricuspid regurgitation, survival, heart failure, and hospitalization are similar in both women and men [180]. 

Aortic valve replacement: The data on sex differences are not univocal. It was reported that being a woman is an independent risk for surgical aortic valve replacement [181] and a more recent study confirms that women have a 13% higher standardized in-hospital mortality rate than men when undergoing transcatheter aortic valve replacement (TAVR) [182]. However, the clinical trial PARTNER, performed on inoperable and high-risk patients undergoing TAVR, shows that 1-year mortality is lower in women than in men even though women have more vascular complications and bleeding [183]. Another study with TAVR, which enrolled women with fewer comorbidities than men, shows that female patients have more vascular side effects, including bleeding and higher residual gradients, while second valve implantations are more frequent in men, but 1-year mortality did not differ among sexes [184].

## 6. Conclusions

Sex and gender differences have been ascertained in prevention and numerous preclinical and clinical settings, but the influence of sex and gender on MDs is still unclear. It is important to know these sex and gender differences because the use of MDs is very common and of high therapeutic value. Ironically, women may receive fewer MDs but can be more injured than men because they are neglected. As a consequence, several sex- and gender-neutral MDs have been produced, but their efficacy and safety for women were discovered after their introduction to the market. Therefore, multifaced innovation that requires an intersectoral lens is necessary, but it is also fundamental to change our minds by overcoming our implicit biases. An intersectional vision allows us to better understand how different factors (including sex, gender, race/ethnicity, age, socio-economic factors, culture, religion, etc.) work together in characterizing each individual, while also offering an opportunity to provide personalized services that meet needs.

To overcome the gap, multiple actions are necessary such as enhanced financial support. For example, it is time to fund women’s prevalent diseases just like male-prevalent diseases, because the latter receive nearly twice the funds [185]. It is also time to support MD designers and producers that develop MDs according to the needs of women and test them in women to analyze their results after sex stratification. 

Incentives for researchers and producers will accelerate the development of sex–gender-based MDs with intersectoral approaches and different technologies (digital health, AI, sensors, and other assistive technologies, ergonomics, and pharmaceutics). Indeed, developing MDs appropriate for both men and women could be a push toward innovation and economic development, because the inclusion of women and minorities requires design, projection, and production on a new basis [186]. 

Regarding the economic reward for overcoming sex and gender gaps, we must remember that the FemTech industry (focused on solutions to reduce the sex and gender health innovation gap) estimates an increase in its growth to USD 50 billion by 2025 [187]. If this is true, overcoming the sex–gender health gap presents various economic advantages. 

Finally, a system of MD vigilance could help us to reduce the occurrence of adverse effects in women if it includes sex- and gender-related factors. MD vigilance should be harmonized over the world. All regulatory agencies, as the FDA [22] has done, should devise a plan for incorporating sex and gender approaches into MD development and control. 

## 7. Take Home Message

This review has highlighted that sex and gender gaps are present in all processes, from ideation to commercialization, and seeks to encourage us to have a greater awareness of the impact of sex and gender on MDs. To do so, the creation of multidisciplinary team which adopts a sex- and gender-sensitive approach to ensure more rigorous sex–gender data in the field of MDs is an essential requirement for the personalization of prevention and care following the principle of the health equity.

## Data Availability

No new data were created or analyzed in this study. Data sharing is not applicable to this article.
